# The Interaction Network Ontology-supported modeling and mining of complex interactions represented with multiple keywords in biomedical literature

**DOI:** 10.1186/s13040-016-0118-0

**Published:** 2016-12-19

**Authors:** Arzucan Özgür, Junguk Hur, Yongqun He

**Affiliations:** 1 Department of Computer Engineering, Bogazici University, 34342 Istanbul, Turkey; 2 Department of Biomedical Sciences, University of North Dakota School of Medicine and Health Sciences, Grand Forks, ND 58202 USA; 3 Unit for Laboratory Animal Medicine, University of Michigan, Ann Arbor, MI 48109 USA; 4 Department of Microbiology and Immunology, University of Michigan, Ann Arbor, MI 48109 USA; 5 Center for Computational Medicine and Bioinformatics, University of Michigan, Ann Arbor, MI 48109 USA; 6 Comprehensive Cancer Center, University of Michigan, Ann Arbor, MI 48109 USA

**Keywords:** Interaction Network Ontology, INO, Literature mining, Gene-gene interaction, Interaction keywords, Interaction types, Gene regulation, SciMiner, LLL dataset

## Abstract

**Background:**

The Interaction Network Ontology (INO) logically represents biological interactions, pathways, and networks. INO has been demonstrated to be valuable in providing a set of structured ontological terms and associated keywords to support literature mining of gene-gene interactions from biomedical literature. However, previous work using INO focused on single keyword matching, while many interactions are represented with two or more interaction keywords used in combination.

**Methods:**

This paper reports our extension of INO to include combinatory patterns of two or more literature mining keywords co-existing in one sentence to represent specific INO interaction classes. Such keyword combinations and related INO interaction type information could be automatically obtained via SPARQL queries, formatted in Excel format, and used in an INO-supported SciMiner, an in-house literature mining program. We studied the gene interaction sentences from the commonly used benchmark Learning Logic in Language (LLL) dataset and one internally generated vaccine-related dataset to identify and analyze interaction types containing multiple keywords. Patterns obtained from the dependency parse trees of the sentences were used to identify the interaction keywords that are related to each other and collectively represent an interaction type.

**Results:**

The INO ontology currently has 575 terms including 202 terms under the interaction branch. The relations between the INO interaction types and associated keywords are represented using the INO annotation relations: ‘has literature mining keywords’ and ‘has keyword dependency pattern’. The keyword dependency patterns were generated via running the Stanford Parser to obtain dependency relation types. Out of the 107 interactions in the LLL dataset represented with two-keyword interaction types, 86 were identified by using the direct dependency relations. The LLL dataset contained 34 gene regulation interaction types, each of which associated with multiple keywords. A hierarchical display of these 34 interaction types and their ancestor terms in INO resulted in the identification of specific gene-gene interaction patterns from the LLL dataset. The phenomenon of having multi-keyword interaction types was also frequently observed in the vaccine dataset.

**Conclusions:**

By modeling and representing multiple textual keywords for interaction types, the extended INO enabled the identification of complex biological gene-gene interactions represented with multiple keywords.

**Electronic supplementary material:**

The online version of this article (doi:10.1186/s13040-016-0118-0) contains supplementary material, which is available to authorized users.

## Background

Extracting the existence of interactions among biomolecules and identifying the types of these interactions are vital for a better understanding of the underlying biological processes and for the creation of more detailed and structured models of interactions such as in biological pathways. One major type of biomolecular interactions is the interactions among genes and proteins. In this article, we use the commonly applied GENETAG-style named entity annotation [1], where a gene interaction involves genes or gene products (proteins).

The types of interactions (or events) among biomolecules are in general signaled with specific interaction keywords (trigger words). For example, the interaction keyword “up-regulates” signals an interaction type of positive regulation, whereas the keyword “inhibits” signals an interaction type of negative regulation. We have previously collected over 800 interaction keywords, which we used with support vector machines (SVM) [2] to classify pairs of genes or proteins as interacting or not [3]. We have also shown that the usage of ontologies, such as the Vaccine Ontology (VO), can enhance the mining of gene-gene interactions under a specific domain, for example, the vaccine domain [3, 4] or vaccine-induced fever domain [5]. These over 800 interaction-associated keywords provide us tags for mining interaction relations between two genes or proteins. However, this is basically a binary result of an interaction between two molecules or entities. In other words, two entities are classified as interacting or not interacting.

To extend from the binary yes/no results, we hypothesized that the ontological classification of interaction-associated keywords would allow us to further identify and classify the types of interactions, consisting of multiple interaction keywords (e.g., *regulation of transcription*). A biological ontology is a set of computer- and human-interpretable terms and relations that represent entities in a biological domain and how they relate to each other [6]. Based on the above hypothesis, we ontologically classified the interaction-related keywords in the Interaction Network Ontology (INO), a community-driven ontology of biological interactions, pathways, and networks [3, 7]. INO classifies and represents different levels of interaction keywords used for literature mining of genetic interaction networks. Its development follows the Open Biological/Biomedical Ontology (OBO) Foundry ontology development principles (*e.g.*, openness and collaboration) [8]. In a recent study, we demonstrated the utility of using INO and a modified Fisher’s exact test to analyze significantly over- and under-represented enriched gene-gene interaction types among the vaccine-associated gene-gene interactions extracted using all PubMed abstracts [7]. Our study showed that INO would provide a new platform for efficient mining and analysis of topic-specific gene interaction networks.

Nevertheless, there still exist two more challenges regarding the INO-based classification method. The first is that the INO-based data standardization is not easy for tool developers to deploy. The second is that current INO-based classification focuses on the classification of interaction types signaled with one keyword in a sentence. However, it is quite frequent that two or more interaction-related keywords collectively signal an interaction type in a sentence. Such combinations of keywords were discussed in the Discussion section of our previous paper without further exploration [7]. In this article, we report our effort to address these two challenges, including the further development and standardization of INO-based classification method and INO-based classification of multiple interaction keywords representing interaction types in sentences. We have also applied these to two case studies of gene-gene interactions in a model bacterium (LLL dataset) and vaccine-related literature.

## Methods

**Fig. 1:**
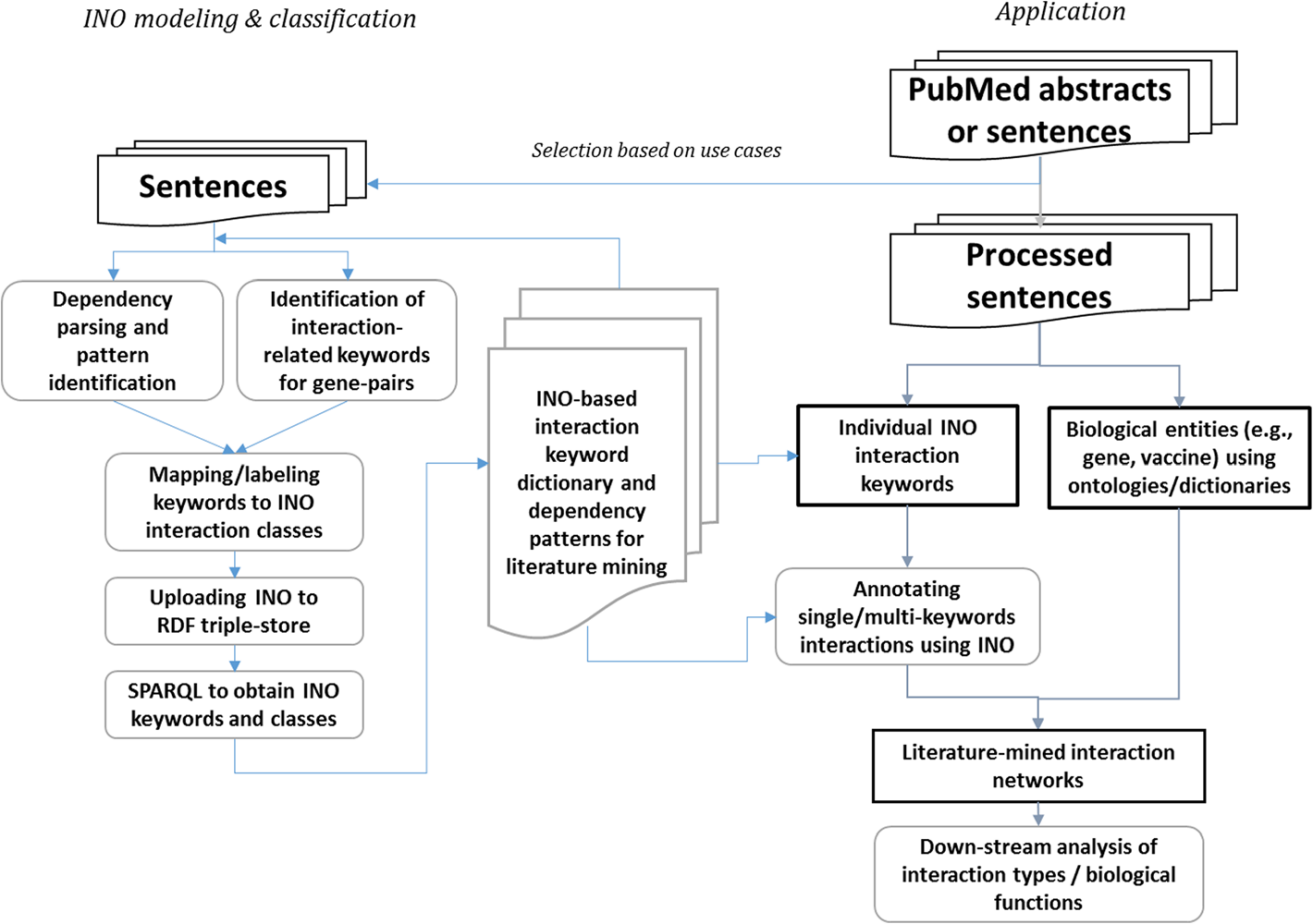
INO modeling and application workflow. This figure illustrates the overall workflow of our approach

Figure 1 illustrates the overall workflow of our proposed approach of the multi-keyword INO modeling and its application in literature mining for gene-interaction analysis. Briefly, the INO modeling procedure (as shown in the left part of Fig. 1) aims at identifying and classifying the interaction patterns of two INO keywords (see the *INO ontology modeling and editing* section below for more details). Once the INO-interaction keyword dictionary is established, it can be applied to constructing interaction networks of biological entities from any set of biomedical literature using SciMiner [7, 9] (as shown in the right part of Fig. 1).

### INO ontology modeling and editing

INO was formatted using the Description Logic (DL) version of the Web Ontology Language (OWL2) [10]. The Protégé OWL Editor [11] was used to add and edit INO specific terms. To identify INO interaction types containing two or more keywords used for literature mining of gene-gene interactions, we manually annotated sentences from selected PubMed abstracts as described later and ontologically modeled each interaction type in INO.

As shown in Fig. 1, sentences with potential multiple interaction keywords (from gold standard sets) were first scanned to identify individual single-word INO keywords and biological entities. For any sentences with two or more interaction keywords identified, combinations of two keywords were queried against the dictionary of keywords associated with existing INO interaction classes. For any two keyword patterns that were not included in the current dictionary, INO experts manually examined the sentences and two-keyword patterns to confirm their valid interactions, updated the INO annotations accordingly with new entries, and uploaded the updated INO to an RDF triple store so that SPARQL could be used to create a new INO keyword dictionary for literature mining.

### Application of INO ontology in literature mining using SciMiner

Using the established INO-interaction keyword dictionary, SciMiner [7, 9], our in-house literature mining tool, was employed to identify biological entities from biomedical literature (Fig. 1). SciMiner accepts PubMed abstracts or sentences as input. After internal preprocessing of the abstracts/sentences, SciMiner identified biological entities such as gene/protein or any ontology terms (e.g. vaccine ontology terms) as well as single-word level INO terms. Sentences with at least two identified entities and one or more INO terms were used in the interaction modeling. Sentences with two interaction keywords can further go through multi-keyword interaction modeling, and a final interaction network can be generated and subjected to down-stream functional analysis.

### SPARQL query of the INO subset of interaction keywords used for literature mining of gene-gene interactions

The Ontobee SPARQL endpoint (http://www.ontobee.org/sparql) was used to obtain the literature mining keywords by querying the INO ontology content stored in the He Group RDF triple store [12]. This triple store was developed based on the Virtuoso system [13]. The data in the triple store can be queried using the standard Virtuoso SPARQL queries.

### OntoFox extraction of an INO subset of interaction terms that can be classified by two or more keywords in one sentence

To better identify the hierarchical patterns of INO terms that were associated with literature mined complex multi-keywords in individual sentences, the OntoFox tool [6] was used to extract a subset of INO containing these directly identified INO terms and the terms related to them.

### Gold standard Learning Logic in Language data analysis

In order to analyze the characteristics of interactions, which are signaled with more than one keywords, we used the gene/protein interaction dataset from the Learning Logic in Language (LLL) Challenge [14]. The LLL dataset contains gene/protein interactions in *Bacillus subtilis*, which is a model bacterium [6]. The dataset contains 77 sentences and 164 pairs of genes/proteins that are described as interacting in these sentences (Additional file 1). We manually annotated the LLL dataset for the interaction types and the keywords that signal them. The annotation was performed by two experts, who reviewed the output of the single-word interaction keywords identified by SciMiner, then carefully examined for multi-keyword interactions. Discrepancy between the two experts was resolved by a third expert.

### Identification of related keywords using dependency parsing

A sentence may contain multiple interaction keywords and multiple gene pairs. In such cases, it is crucial to determine the set of related keywords that in combination represent an interaction type. We can take the following sentence “The expression of *rsfA* is under the control of both *sigma(F)* and *sigma(G)*.” from the LLL dataset as an example. The sentence describes an interaction between the gene pairs *rsfA*-*sigma(F)* and *rsfA*-*sigma(G)*. There are two interaction keywords: “expression” and “control”. It is important to determine that these two keywords do not individually represent an interaction, but are associated with each other in the sentence and together signal the interaction type of “regulation of expression”. Two keywords may be associated with each other, even if they are not close to each other in the sentence. For example, in the sample sentence “expression” and “control” are five words apart from each other.

**Fig. 2:**
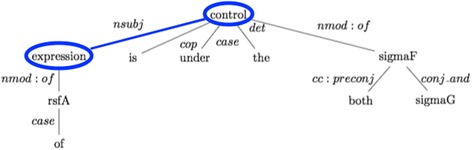
Example dependency parse tree with direct connection between two related keywords. The figure illustrates the dependency parse tree of a sentence “The expression of rsfA is under the control of both sigma(F) and sigma(G)” obtained from the LLL dataset. Dependency parsing was done using Stanford Parser. The related keywords “expression” and “control” are directly connected to each other

The dependency tree representations of sentences, which model the grammatical relations (*e.g.*, subject, object, and modifier) among the words in a sentence, are in general useful to capture such long distance relations among words. We analyzed the dependency parse trees of the sentences in the LLL dataset and identified dependency patterns for related pairs of keywords. Figure 2 shows the dependency parse tree (universal dependencies enhanced representation) for the sample sentence obtained by using the Stanford Parser, which is an open-source NLP library for text processing [15]. The interaction keywords “expression” and “control” are directly connected to each other with the dependency relation type nominal subject (nsubj). In other words, “expression” is the nominal subject of “control”. We considered the pairs of keywords and identified them as associated (*i.e.*, represent an interaction type in combination), if they are directly connected with a dependency relation.

### Vaccine gene-gene interaction literature mining use case

In our previous studies, we used ontology-based SciMiner to extract and analyze gene-gene interactions in the vaccine domain using all PubMed abstracts [7]. In this study, we further annotated those sentences, including two or more interaction-related keywords for annotating gene-gene interactions. The results were then systematically analyzed.

## Results

### INO representation of complex interaction types

As defined previously, INO is aligned with the upper-level Basic Formal Ontology (BFO) [8]. In INO, a biological interaction is defined as a processual entity that has two or more participants (*i.e.*, interactors) that have an effect upon one another. To support ontology reuse and data integration, INO imports many terms from existing ontologies [7], such as the Gene Ontology (GO) [16], and PSI Molecular Interactions (PSI-MI) [17]. As of September 25, 2016, INO has 575 terms, including 156 terms with INO prefix and 419 terms imported from 13 other ontologies (http://www.ontobee.org/ontostat/INO). The INO interaction branch contains 202 ontology classes.

**Fig. 3:**
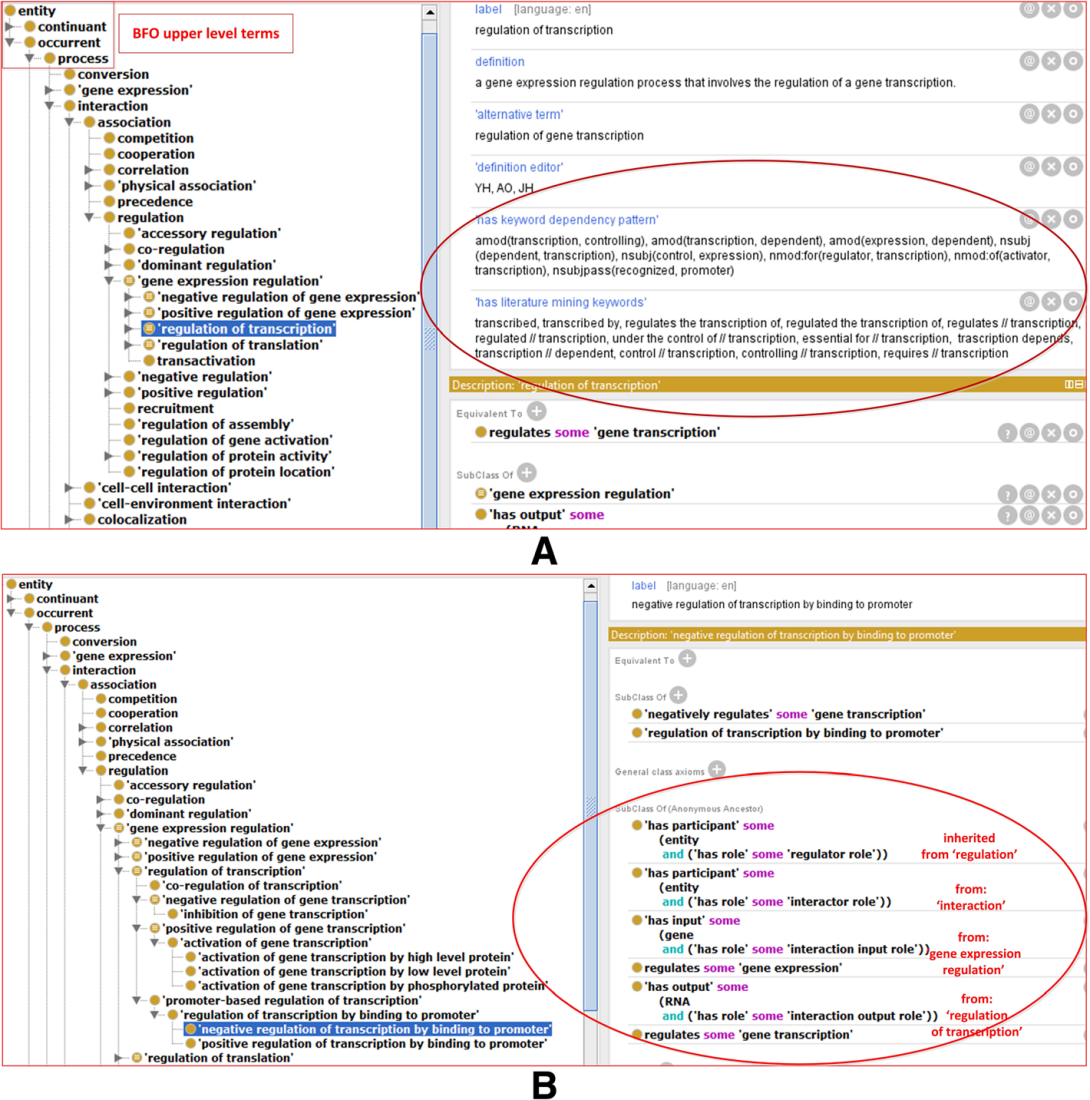
INO representation of interaction types. **a** INO representation of ‘regulation of transcription’. Equivalent and subclass axioms are defined for this class. As shown in the figure, INO is aligned with BFO as its upper level ontology. The annotated literature mining keywords and keyword dependency patterns for the INO class are highlighted with oval circle. **b** INO representation of ‘negative regulation of transcription by binding to promoter’. In addition to its subclass definitions, this INO terms also inherits many axioms defined in different levels of its ancestor terms

In the present study, we focused on the branch of gene-gene regulation, particularly gene expression regulation (Fig. 3). For the INO term ‘gene expression regulation’, the input interactor is a gene, the output interactor is a gene product including a RNA or protein, and the regulator is typically a protein. Therefore, the term ‘gene expression regulation’ represents that the regulator regulates the expression of a gene into a RNA (called transcription) or a protein (called expression). To semantically represent the information, the equivalent class definition of this term ‘gene expression regulation’ is: *regulates some ‘gene expression’*. A subclass necessary condition definition of this term is: *‘has input’ some (gene and (‘has role’ some ‘interaction input role’))*.

There exist different subtypes of ‘gene expression regulation’, for example, ‘positive or negative regulation of gene expression’, and ‘regulation of transcription (or translation)’. Figure 3a shows an example of how INO defines the term ‘regulation of transcription’. In addition to its text definition, INO also generates many logic axioms. An equivalent class definition of the term is defined: *regulates some ‘gene transcription’*, where ‘regulates’ is an object property (or called relation) and ‘gene transcription’ is a gene expression process that transcribes a gene to RNA. In addition to asserted axioms, many axioms are also inherited from its parent term ‘gene expression regulation’ (Fig. 3a).

Various subtypes of ‘regulation of transcription’ exist. For example, there are different subtypes of positive or negative regulation of transcription. One commonly seen subtype of regulation of transcription is via a promoter. A promoter is a region of DNA located near the transcription start site of a gene, and the binding between a promoter sequence and a transcription factor is required to initiate a transcription. Such a binding may positively or negatively regulate the transcription. Therefore, Fig. 3b shows the INO term ‘negative regulation of transcription by binding to promoter’. This term includes a subclass definition: *‘negatively regulates’ some ‘gene transcription’*. In addition, it also includes many axioms inherited from different levels of ancestor terms, including ‘regulation of transcription by binding to promoter’, ‘regulation of transcription’, ‘gene expression regulation’, ‘regulation’, and ‘interaction’ (Fig. 3b). Such hierarchical inheritance of axioms is an advantage of the ontology strategy for computer-assisted automated reasoning.

### Standard INO representation of literature mining keywords for interaction terms

In this section, we introduce how INO is used to represent the complex interaction types that match two or more keywords in individual sentences from biomedical literature.

Different gene-gene interaction types exist from biomedical literature. Some gene-gene interactions are characterized with a single interaction keyword. For example, in the sentence “Dephosphorylation of SpoIIAA-P by SpoIIE is strictly dependent on the presence of the bivalent metal ions Mn^2+^ or Mg^2+^” [18], the type of interaction between SpoIIAA-P and SpoIIE is dephosphorylation reaction, which is characterized with the interaction keyword “dephosphorylation”. On the other hand, there are also more complex interactions that are characterized with two or more interaction keywords. For example, the phrase of a sentence “*sigmaB*- and *sigmaF*-dependent promoters of *katX*” [19] indicates that *sigmaB* and *sigmaF* regulate *katX* through the *katX* promoters. Therefore, the interaction illustrated in this phrase is an instance of the INO interaction type ‘promoter-based regulation of transcription’.

Consider the sentence “In the mother cell compartment of sporulating cells, expression of the *sigE* gene, encoding the earlier-acting sigma factor, sigmaE, is negatively regulated by the later-acting sigma factor, sigmaK” [20]. The relation between the *sigE* and *sigmaK* genes is characterized with the interaction keywords “expression” and “negatively regulated”. The type of relation is INO term of ‘negative regulation of gene expression’ (INO_0000039). SigmaK negatively regulates the expression of *sigE*. Such relations are represented as complex events in the Genia event corpus [21] used in the BioNLP Shared Tasks, where the expression of *sigE* is considered as the first event and the negative regulation of this event by the *sigmaK* gene is considered as the second event. In contrast, INO represents such complex events using a different strategy as described below.

As shown in Fig. 3, the literature mining keywords for an INO term are defined as an annotation using the annotation property ‘*has literature mining keywords*’. To provide a reproducible strategy of representing the literature mining keywords, we used the sign “//” to separate two keywords, which indicates that these two keywords do not have to be next to each other in a sentence (Fig. 2). For example, multiple keywords are added for the INO term ‘regulation of transcription’ (INO_0000032), including *“transcription//dependent, regulated//transcription, requires//transcription”.* These expressions mean that the two keywords such as “requires” and “transcription” can be separate in one sentence, for example, “sspG transcription also requires the DNA binding protein GerE” [22].

Another annotation property: ‘*has keyword dependency pattern*’ (Fig. 3a) specifies the dependency pattern of the literature keywords that match to the ontology interaction type. For example, the INO term ‘regulation of transcription’ has many associated keyword dependency patterns such as amod(transcription, controlling), amod(transcription, dependent), amod(expression, dependent), and nsubj(control, expression) (Fig. 3a). Table 1 provides five keyword dependency patterns and their examples. These patterns are frequently identified in the sentences representing gene-gene interaction types.

**Table 1:** Five keyword dependency patterns and examples

Relation Type	Explanation	Dependency pattern example	Sample sentence
nsubj(A,B)	B is nominal subject of A	nsubj(control, expression)	The expression of rsfA is under the control of both sigma(F) and sigma(G)
nsubjpass(A, B)	B is passive nominal subject of A	nsubjpass(recognized, promoter)	The ald promoter, like the sigE promoter, is believed to be recognized by sigmaA RNA polymerase, suggesting that sigmaK may inhibit sigmaA activity late in sporulation.
dobj(A, B)	B is direct object of A	dobj(inhibit, activity)	The ald promoter, like the sigE promoter, is believed to be recognized by sigmaA RNA polymerase, suggesting that sigmaK may inhibit sigmaA activity late in sporulation.
amod(A,B)	B is adjectival modifier of A	amod(transcription, GeneX-dependent)	These results demonstrate that sigmaK-dependent transcription of gerE initiates a negative feedback loop in which GerE acts as a repressor to limit production of sigmaK.
nmod(A,B)	B is nominal modifier of A	nmod(essential, expression)	Both SigK and GerE were essential for ykvP expression, and this gene was transcribed from T5 of sporulation.

### SPARQL retrieval of INO interaction types and associated keyword terms for literature mining of gene-gene interactions

INO is represented using the Web Ontology Language (OWL) [10] format. The contents of the OWL files can be expressed with Resource Description Framework (RDF) triples and stored in an RDF triple store database. The RDF data model makes statements about resources in the form of subject-predicate-object expressions (*i.e.*, triples). SPARQL (a recursive acronym for SPARQL Protocol and RDF Query Language) [23] can be used to retrieve data stored in a RDF triple store. The INO ontology content has been deposited in the Hegroup RDF Triple Store [12], which is the default RDF triple store for the ontologies in the Open Biological and Biomedical Ontologies (OBO) library (http://www.obofoundry.org/). After the ontology is stored in the RDF triple store, the INO ontology information can be queried using the Ontobee SPARQL query interface (http://www.ontobee.org/sparql).

**Fig. 4:**
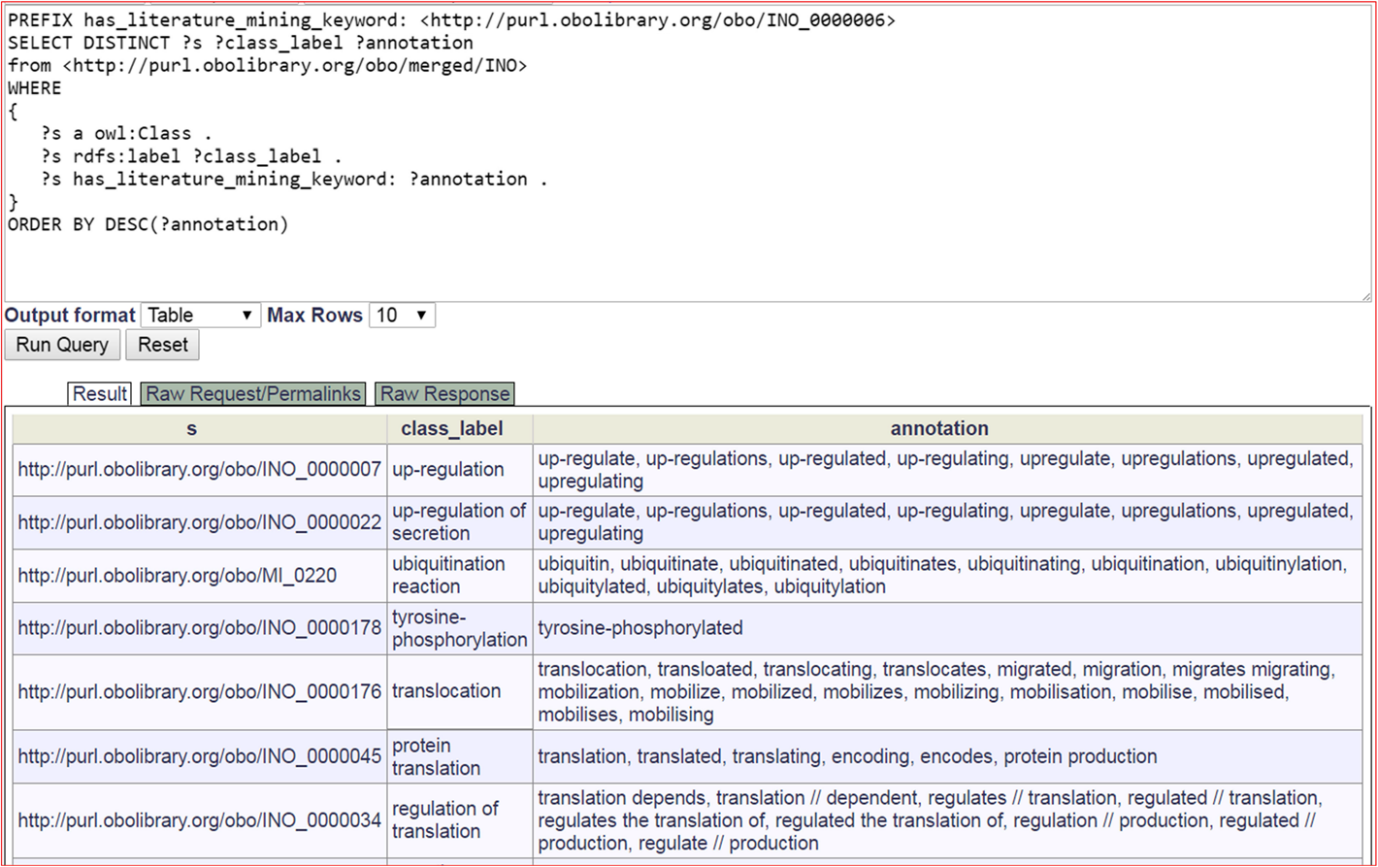
SPARQL query of interaction keywords for INO interaction class terms. This query was performed using the Ontobee SPARQL query website (http://www.ontobee.org/sparql/). This figure is a screenshot of the SPARQL code and a portion of the results

SPARQL provides a quick and efficient way to obtain the INO literature mining keywords and associated interaction types. Figure 4 shows the usage of a SPARQL query to automatically generate the INO subset for literature mining. Each row of the SPARQL query includes the URI of an INO ontology interaction term, the label of the interaction type, and the keyword annotations as represented by the annotation property ‘has literature mining keywords’ and ‘has keyword dependency pattern’ (Fig. 3). The information can then be downloaded, saved in Excel, and used for literature mining in a software program such as SciMiner as described below.

### Incorporation of INO literature mining system to a software program

SciMiner is our in-house literature mining software program for identifying interactions among genes/proteins/vaccines and analyzing their biological significance [9]. We recently incorporated INO into SciMiner and demonstrated its successful application to the identification of specific interaction types significantly associated with gene-gene interactions within the context of vaccine [7]. SciMiner can also be utilized in identifying and modeling two interaction keywords, which will be eventually used to improve the final literature-mined interaction network.

### Identification of related keywords in the LLL dataset using dependency patterns

Our primary dataset in this study was the LLL dataset, the gene-gene interactions of which were analyzed and the dependency patterns for the interaction types represented with two interaction keywords are obtained by using the Stanford Parser [15]. Two keywords directly connected by a dependency relation are considered as associated with each other. The dependency patterns as well as the sentences are summarized in Table 1. Out of the 107 interactions in the LLL dataset represented with two-keyword interaction types, 86 related keyword pairs were identified by using the direct dependency relations. In the remaining 21 interactions, the related keywords were not directly connected with a dependency relation, but were rather indirectly connected.

**Fig. 5:**
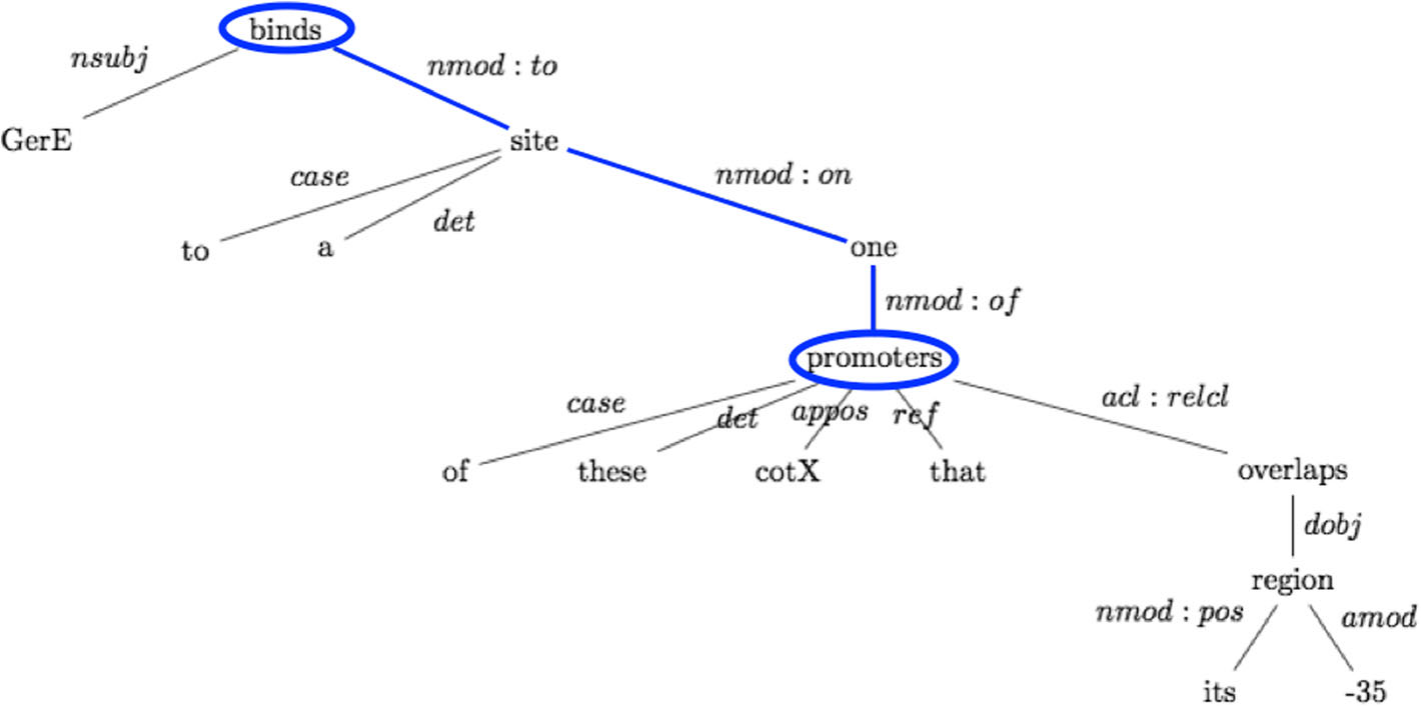
Example dependency parse tree with indirect connection between two related keywords. The dependency parse tree for the sample sentence “GerE binds to a site on one of these promoters, cotX, that overlaps its −35 region.” The related interaction keywords “binds” and “promoters” are not directly connected to each other with a dependency relation

Figure 5 provides an example of such indirect dependency relation. In the sentence “GerE binds to a site on one of these promoters, cotX, that overlaps its −35 region”, the interaction keywords “binds” and “promoters” collectively represent the interaction type “regulation of transcription by binding to promoter”. However, as shown in Fig. 5, there is no a direct dependency relation between these keywords. Identifying such indirectly connected pairs of related keywords requires further investigation.

### Annotation of the LLL dataset for interaction types

Given a sentence and the interacting pair of proteins/genes, we annotated the type of relation between them and the interaction keywords signaling this relation. The annotation was done by two human experts independently. Out of 164 interactions, 26 interactions had conflicts in the interaction keywords and 13 interactions had conflicts in the interaction type (INO Type), which were resolved by a third human expert (see Additional file 1 for the details). Our interaction type and keyword annotation of the dataset is available in Additional file 1. As an example, consider the sample sentence “Transcriptional studies showed that nadE is strongly induced in response to heat, ethanol and salt stress or after starvation for glucose in a sigma B-dependent manner” [24] from the LLL dataset. The interacting protein/gene pairs (*e.g.*, nadE and sigma B) have already been annotated in the dataset. The type of interaction between nadE and Sigma B is “positive regulation of gene transcription”, in other words Sigma B positively regulates the transcription of nadE. The relevant interaction keywords are “transcriptional”, “induced”, and “dependent”.

**Fig. 6:**
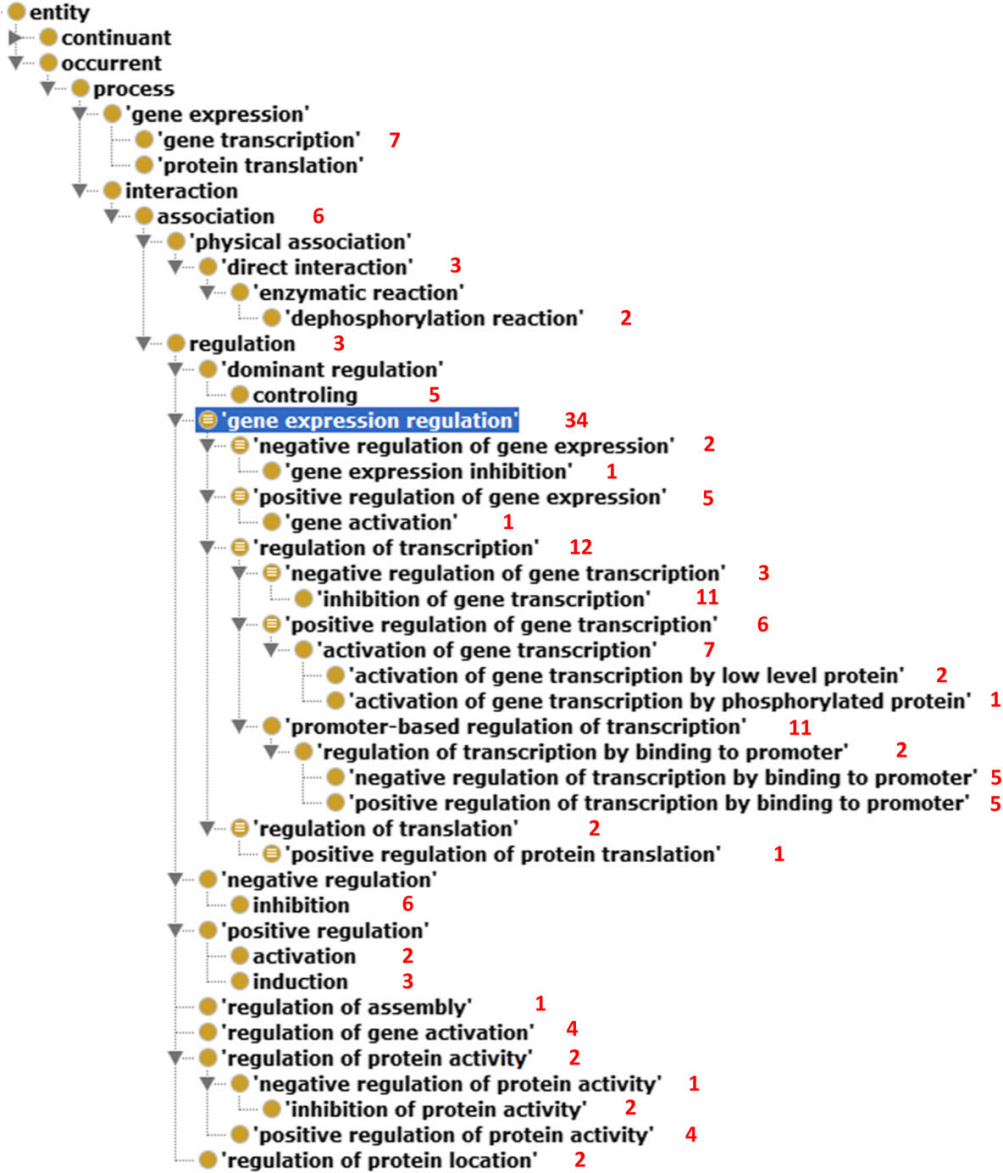
Hierarchical display of interaction classes found in the LLL dataset. This figure illustrates the hierarchical display of 34 interaction classes and the numbers of sentences associated with these classes in the LLL dataset. OntoFox was used to generate the INO subset, and the Protégé OWL editor was used to visualize the hierarchical structure

Our annotation of the LLL dataset for interaction types showed that many regulatory relations between gene/protein pairs are represented with multiple keywords. While the interactions among 42 pairs of genes/proteins were represented with a single keyword, the interactions among 122 pairs were signaled using multiple keywords. These interactions correspond to 34 different classes of *regulation* in INO. Figure 6 shows the hierarchical structure of these 34 classes, their related classes, and the number of gene/protein pairs in the sentences identified for each class.

Our study of the LLL dataset indicated that the majority of the sentences are related to the gene expression regulation, especially in the area of transcriptional regulation. More sentences describe positive regulation rather than negative regulation. An interesting observation is the presence of many sentences focusing on the domain of promoter-based regulation of transcription (Fig. 3). In addition to gene expression regulation, this dataset also includes other types of gene regulation, for example, regulation of protein location, regulation of gene activation, and regulation of protein activity. It is noted that protein activity is different from gene expression. Protein activity depends on many factors other than expression, such as correct folding of the protein and the presence of any required cofactors.

Our analysis showed that most multi-keyword interactions are represented with two keywords. Consider the interaction between KinC and Spo0A ~ P in the sentence “KinC and KinD were responsible for Spo0A ~ P production during the exponential phase of growth in the absence of KinA and KinB” [25]. This sentence states that KinC is responsible for Spo0A ~ P production. The interaction type between these genes is classified as “regulation of translation” in INO. The two keywords signaling this interaction are “responsible” and “production”. The keyword “responsible” signals that this is an interaction of type “regulation”, whereas the keyword “production” signals that this is a specific type of regulation, namely “regulation of translation”. We can consider “responsible” as the main type signaling keyword and “production” as the secondary (sub) type signaling keyword.

There are also more complex interactions, which are represented with more than two keywords. For example, in the sentence “A low concentration of GerE activated cotB transcription by final sigma(K) RNA polymerase, whereas a higher concentration was needed to activate transcription of cotX or cotC.” [26], the interaction between GerE and cotB is signaled with the three keywords “low concentration”, “activated”, and “transcription”. The type of interaction corresponds to the INO class “activation of gene transcription by low level protein”. In another sentence “sigmaH-dependent promoter is responsible for yvyD transcription” [27], four keywords are used: “dependent”, “promoter”, “responsible”, and “transcription”. Such a complex interaction is labeled as “promoter-based regulation of transcription” in INO.

### Analysis of vaccine-based gene-gene interaction literature mining results

Our previous INO-based literature mining study used an INO-based SciMiner program to identify the gene-gene interactions in the vaccine domain using all PubMed abstracts [7]. To identify the level of multi-keyword interaction types in the vaccine-domain literature, we manually examined randomly selected 50 sentences identified by SciMiner, a portion of the whole vaccine corpus. Our results suggested that similar to the LLL dataset, over 50% of sentences use two or more keywords to represent specific gene-gene interaction types. Since this paper focuses on the research domain of how to apply ontology for multi-keyword interaction literature mining instead of the science behind the vaccine domain, we did not investigate deeply into the vaccine corpus.

## Discussion

In this paper, we investigated the interaction types that are characterized with multiple keywords used in combination. The main contributions are: (1) Extending INO by modeling interaction types (classes) each signaled with multiple keywords in literature sentences and adding many new terms by analyzing the LLL and vaccine datasets, (2) Standardizing INO-based literature mining for easy use and testing by future studies. (3) Characterizing and demonstrating multi-keyword interaction type ontology modeling of literature sentences by analyzing the LLL and vaccine-gene interaction datasets.

Ontology-based Literature Mining (OLM) is an emerging research field that applies ontology to support literature mining. With the support of ontologies, OLM significantly enhances literature mining performance [28–35]. For example, the Gene Ontology (GO) has been used in supporting literature mining [29, 30, 32]. The NCBO BioPortal Annotator [31] is a web service that supports ontology-based tagging that uses Mgrep [36] as the concept recognizer tool [37]. We have effectively applied OLM in mining gene-gene interactions [3–5, 7, 38]. We have also developed a VO-based SciMiner method to mine the interactions among vaccines and genes [3]. In this study, based on our observation of the frequent usage of multiple keywords for one specific interaction type [7], we extended our previous ontology-based gene-gene interaction research to focus on ontological representation and modeling of this special type of gene-gene interactions and multi-words associated with these interaction types. It is noted that an early version of this study was reported in the International Workshop on Biomedical Data Mining, Modeling, and Semantic Integration (BDM2I2015) in the International Semantic Web Conference (ISWC 2015) [38]. The current peer-reviewed journal article has significantly extended the early proceeding paper.

Literature mining methods for extracting interactions among biomedical entities including genes and proteins typically formulate the problem as a binary classification task, where the goal is to identify the pairs of entities that are stated to interact with each other in text [39, 40]. Several different methods have been proposed to tackle this problem ranging from relatively simpler co-occurrence based methods [41] to more complex methods that make use of the syntactic analysis of the sentences [42–44], mostly in conjunction with machine learning methods [45–47].

Multi-keyword interactions have been represented as complex events in the Genia corpus [21], which has also been used in the BioNLP Shared Tasks on Event Extraction. In this representation, in order to identify the complex events, first the simple events (e.g. gene expression, regulation) signaled with individual keywords need to be identified. Next, the simple events are combined to form a complex event. For instance, given a sentence that states that gene A regulates the expression of gene B, the expression of gene B is represented as Event 1 (i.e., expression of gene B), and Event 2 is a complex event where gene A regulates Event 1. Therefore, we could infer a possible relation between gene A and gene B, by the association of Event 1 – gene B – Event 2 – gene A. Such recognition of the gene A-B interaction is indirect, and may become even more complex when multiple events (with multiple keywords) are applied. Compared to the Genia approach, INO provides a more fine-grained and direct classification of interaction types and can directly model the relation between two biomolecules (*e.g.*, genes or proteins). For instance, the interaction between gene A and gene B in the above example is directly modeled as the interaction type “regulation of gene expression” in INO.

As a conceptual model for the domain of gene regulation, the Gene Regulation Ontology (GRO) [48] models complex gene regulatory events similarly to INO. GRO has recently been used in the Corpus Annotation with Gene Regulation Ontology Task in the 2013 edition of BioNLP Shared Task [49]. The domains of GRO and INO differ. GRO focuses on only gene regulations. However, INO targets the broader scope of interactions and interaction networks. Similar to INO, GRO is also aligned with the Basic Formal Ontology (BFO) and many other ontologies such as the Gene Ontology (GO). However, for the ontology alignments, GRO uses its own identifiers and references back to the original ontologies; in contrast, INO directly imports related terms from other ontologies. Technical representations of entities in INO and GRO also differ in many aspects. Compared to GRO, one of the main advantages of INO is that the interaction types and sub-types are associated with manually compiled comprehensive lists of literature mining keywords and dependency patterns.

These keywords and patterns can be incorporated in dictionary-based or statistical taggers for tagging the interaction keywords in text, which can then be used to map the interactions to their corresponding types in INO. Using the dependency parse trees of the sentences, we proposed an approach for identifying interaction keyword pairs that together represent an interaction type in INO. We showed that the majority of the related keyword pairs in the LLL dataset are directly connected to each other with a dependency relation. However, the remaining-related keywords (19 cases out of 89) do not have direct dependency relations with each other (Fig. 5). In addition, there are complex interactions, which are signaled with more than two keywords. As future work, we will investigate generating complex dependency patterns for these types of interactions.

Future work includes automatic identification and modeling of novel multi-keyword interactions by SciMiner. The currently available multi-keyword interactions were manually identified by experts, who reviewed individual cases of multiple INO keywords in the same sentence. An automated machine learning-based approach to identify such multi-keyword interactions will be developed and incorporated into INO and SciMiner. In addition to the identification of multi-keywords in the same sentence, we are expanding our ontology-based mining approach to identify interactions across multiple sentences. The complete standalone pipeline will be available upon completion of the development.

In order to ontologically represent and to efficiently identify these complex interaction types across multiple sentences, we plan to standardize them using a regular expression-based approach in addition to the notion of the current ‘//’-based and dependency pattern based strategy. This will be implemented by referencing the strategy in the Stanford TokensRegex Framework [50]. It is possible to extend the INO dependency patterns by incorporating the regular expression-based representations in the Stanford TokensRegex Framework. Such a strategy can be added as an important INO attribute so that other literature mining community members can use them in their own applications.

In this paper, we demonstrated our strategy of integrating INO with the SciMiner tagger for ontology-based literature mining. Currently, the integrated INO-SciMiner works as a standalone package; and it can be easily incorporated into other literature mining pipelines, if desired. The current SciMiner system can identify gene/protein and vaccine, but will be updated to be able to identify other entities such as drug, tissue, and *etc*., thus, the future version of INO-integrated SciMiner can be applied to not only the typical gene-gene interaction, but also other interactions such as gene-drug interaction, drug-chemical, drug-tissue and various types of interaction.

## Conclusions

The Interaction Network Ontology (INO) is extended with a specifically defined annotation property to model and represent two or more textual keywords that are used to represent specific molecular interaction types. A SPARQL query is able to easily extract the information of complex interactions and corresponding keywords. Our LLL and vaccine use cases demonstrate the frequent occurrence of such complex keyword patterns in biomedical literature and our INO-based strategy supports the modeling and analysis of these complex interaction types.

## Additional file

Additional file 1: Interaction keywords and INO type annotations for the LLL data set. This additional file includes annotations﻿ of the LLL dataset with INO multiple keyword interaction types. (XLSX 29 kb)

## Abbreviations

BFO: Basic formal ontology
GO: Gene ontology
INO: Interaction Network Ontology
NCBO: National Center for Biomedical Ontology
PSI-MI: Proteomics Standards Initiative-Molecular Interaction
SVM: Support vector machine
VO: Vaccine ontology
